# Physicochemical Characterization and Solar-Driven Chromophore Removal of Hydroalcoholic *Larrea tridentata* Extracts in Aqueous Media

**DOI:** 10.3390/molecules31162861

**Published:** 2026-08-16

**Authors:** David Quiroz-Cardoza, Juan C. Pantoja-Espinoza, José Béjar, José C. Espinoza-Hicks, Ma. Rosario Peralta-Pérez, Antonino Pérez-Hernández, Francisco Paraguay-Delgado, Víctor M. Orozco-Carmona

**Affiliations:** 1 Centro de Investigación en Materiales Avanzados SC (CIMAV), Av. Miguel de Cervantes 120, Chihuahua 31136, Chihuahua, Mexico; david.quiroz@cimav.edu.mx (D.Q.-C.); eduardo.garcia@cimav.edu.mx (J.B.); antonino.perez@gmail.com (A.P.-H.); francisco.paraguay@cimav.edu.mx (F.P.-D.); victor.orozco@cimav.edu.mx (V.M.O.-C.); 2 Faculty of Chemical Sciences, Autonomous University of Chihuahua, University Circuit S/N, Campus UACH II, Chihuahua 31125, Chihuahua, Mexico; jhicks@uach.mx (J.C.E.-H.); mperalta@uach.mx (M.R.P.-P.)

**Keywords:** bioherbicide, herbicide, H_2_O_2_, *Larrea tridentata*, photocatalysis, sunlight, TiO_2_, ZnO

## Abstract

Although plant-derived bioactive compounds are promising sustainable alternatives to synthetic herbicides, their environmental fate and transformation behavior remain poorly understood. This study evaluates the physicochemical stability and solar-driven transformation of phenolic and other complex phytochemical constituents in *Larrea tridentata* hydroalcoholic extracts (AELT) in aqueous media. Spectroscopic and thermal characterization (TGA/DTG, FTIR, ^1^H/^13^C NMR, and GC–MS) revealed a chemically complex phytochemical matrix containing aromatic, phenolic, terpenoid, fatty-acid-derived and other oxygenated constituents, which exhibit a characteristic absorption maximum at 280 nm. Solar-driven oxidation experiments were conducted using TiO_2_, ZnO, and H_2_O_2_. Among the evaluated systems, Solar/ZnO emerged as the most efficient standalone treatment, achieving 89.43% chromophore removal and 88.56% reduction of chemical oxygen demand (COD) after 60 min. The findings demonstrate a clear relationship between the phenolic and other complex phytochemical constituents’ nature of the extract and its high susceptibility to ZnO-assisted solar photocatalysis, which effectively transforms UV-absorbing constituents and reduces the oxidizable organic load. This work establishes a robust framework for assessing the solar treatment of complex phytochemical matrices, supporting the potential application of ZnO-assisted processes for mitigating the organic load of plant-derived formulations in water bodies.

## 1. Introduction

The increasing demand for agricultural productivity has promoted the extensive use of synthetic herbicides for weed control and crop protection [1]. Among these compounds, glyphosate-based formulations remain one of the most widely employed agrochemicals worldwide due to their broad-spectrum activity [2]. However, their continuous application has raised environmental concerns associated with environmental persistence, potential effects on non-target organisms, and the development of herbicide-resistant species [2,3,4]. As a result, increasing attention has been directed toward the development of alternative weed management strategies based on natural products and plant-derived bioactive compounds [5].

Plant extracts with allelopathic activity have emerged as promising candidates for sustainable agricultural applications due to their phytotoxic and bioherbicidal properties [6,7,8,9,10]. Nevertheless, despite the growing interest in plant-derived bioactive formulations, limited attention has been paid to their environmental fate after application [11]. Previous studies have extensively investigated the transformation of individual phytochemicals, including caffeic acid, under photocatalytic [12] and solar-driven conditions [13]. However, the transformation behavior of chemically heterogeneous, multi-component plant-derived matrices may differ from that of individual compounds due to interactions among constituents and matrix effects [14].

*Larrea tridentata* (*L. tridentata*), commonly known as creosote bush, is a perennial plant distributed in arid and semi-arid regions of northern Mexico and the southwestern United States. The species is characterized by a high content of secondary metabolites, including lignans, flavonoids, terpenoids, and phenolic compounds, which exhibit antioxidant, antimicrobial, and phytotoxic activity [15,16,17,18,19]. Among these, nordihydroguaiaretic acid (NDGA) is the most abundant lignan-type phenolic constituent [17].

Advanced oxidation processes (AOPs) have been extensively investigated for the degradation of organic contaminants in water due to their ability to generate highly reactive oxygen species that oxidize complex organic molecules [20,21,22,23]. Semiconductor-based photocatalysis employing TiO_2_ and ZnO has demonstrated high efficiency in degrading dyes, pharmaceuticals, and other persistent organic pollutants under UV and solar irradiation [24]. Likewise, the addition of H_2_O_2_ has also been investigated as a means of modifying oxidative pathways and enhancing photocatalytic treatment under suitable conditions [21,25]. To the best of our knowledge, a comparative assessment of TiO_2_-, ZnO-, and H_2_O_2_-assisted solar treatment of *L. tridentata* aqueous hydroalcoholic extracts (AELT) has not yet been reported.

In this context, the present work addresses this knowledge gap by integrating a multi-technique physicochemical characterization with a comparative evaluation of solar-driven treatment systems. The study aims to evaluate the transformation of UV-active chromophores and the reduction of the oxidizable organic load (COD) of AELT under natural solar irradiation using direct photolysis, H_2_O_2_-assisted, TiO_2_-based, ZnO-based, and combined semiconductor/H_2_O_2_ systems.

## 2. Results

### 2.1. Hydroalcoholic Extraction Yield

A hydroalcoholic extraction yield of 26.36% was achieved, corresponding to 13.18 ± 1.47 g of crude extract recovered from 50 g of dried biomass. The extraction yield indicates that a considerable fraction of extractable constituents was recovered from the plant material under the selected operating conditions. The extraction efficiency can be influenced by several factors, including the extraction technique, solvent composition, extraction time, temperature, and solid-to-liquid ratio. In the present study, the use of a hydroalcoholic solvent system combined with Soxhlet extraction provided continuous solvent recirculation and prolonged contact between the solvent and biomass, favoring the recovery of soluble constituents from *L. tridentata*.

The extract obtained under these conditions was subsequently used for physicochemical characterization and for the evaluation of solar-driven advanced oxidation processes.

### 2.2. Characterization of Biomass and Hydroalcoholic Extract

#### 2.2.1. Proximate and Thermogravimetric Analyses (TGA/DTG)

The proximate analysis results for the raw *L. tridentata* biomass (LT) and the corresponding hydroalcoholic extract (ELT) are summarized in Table 1. Both materials exhibited low moisture contents, with values of 3.08% and 1.90% for LT and ELT, respectively. The reduced moisture content can be attributed to the drying and solvent removal procedures employed during sample preparation, although a minor fraction of retained or reabsorbed water may remain associated with the materials.

The volatile solid (VS) content reached 91.51% for LT and 88.26% for ELT, indicating that both materials were predominantly composed of organic matter. Although the extraction yield averaged 26.36% ± 2.94 relative to the original biomass, the high VS content observed for ELT suggests that a substantial proportion of the extract consisted of organic constituents recovered during the hydroalcoholic extraction process. In contrast, ash contents of 3.82% and 2.21% were determined for LT and ELT, respectively, reflecting the presence of mineral components that remained as residual material after thermal decomposition.

**Figure 1:**
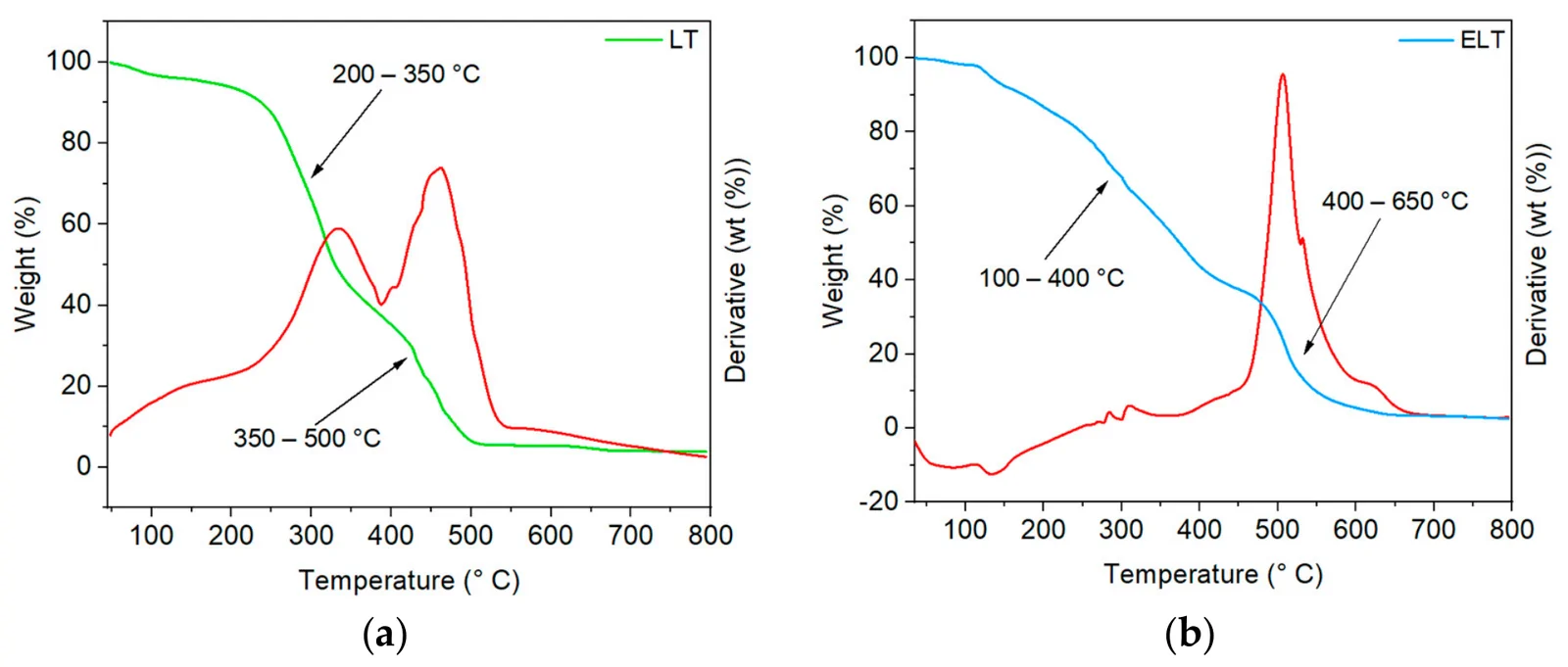
TG and DTG (red) curves of (**a**) raw *L. tridentata* biomass (LT) and its (**b**) hydroalcoholic extract (ELT).

To further investigate the thermal behavior of LT and ELT, thermogravimetric analyses were performed, and the corresponding TG/DTG profiles are presented in Figure 1. The TG profile of LT (Figure 1a) exhibited four main stages of mass loss. The first stage, occurring between approximately 40 and 100 °C, was associated with the removal of physically adsorbed and residual moisture.

The second stage, between 200 and 350 °C, accounted for a weight loss of approximately 36.5% for LT, which is commonly attributed to the decomposition of carbohydrate-rich constituents in plant biomass. The third stage, extending from approximately 350 to 500 °C, exhibited the highest thermal chromophore removal rate with a mass loss of 38%, showing two distinct DTG maximum peaks at 345 °C and 460 °C. This stage was attributed to the decomposition of more thermally resistant organic structures, such as cellulose and complex phenolics. A similar multistep thermal chromophore removal pattern was observed for ELT (Figure 1b). Following the initial dehydration stage (1.90% mass loss up to 150 °C), a gradual weight loss of 28.1% was recorded between 150 and 400 °C. However, the corresponding DTG profile exhibited a dominant chromophore removal event centered within the 450–600 °C interval, reaching its maximum decomposition rate at 512 °C with a major sharp weight loss of 55.4%. This behavior suggests compositional differences between LT and ELT resulting from the hydroalcoholic extraction process, where the prominent peak in ELT reflects the volatilization and pyrolysis of concentrated bioactive phenolic and lignan frameworks. The lower ash content observed for ELT (2.21% at 800 °C) further supports the selective recovery of extractable organic compounds while reducing the relative contribution of inorganic components.

#### 2.2.2. Fourier Transform Infrared Spectroscopy (FTIR) Analysis

**Figure 2:**
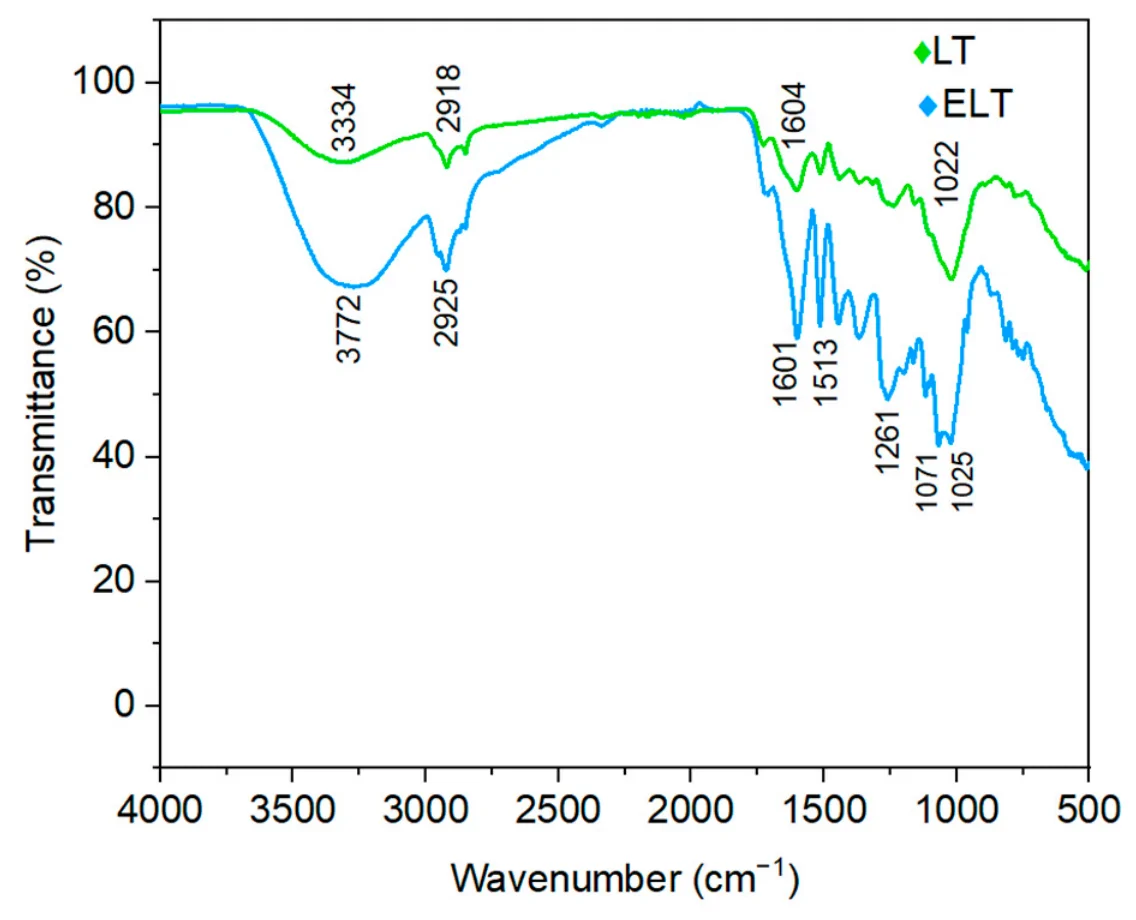
FTIR spectra of raw *L. tridentata* biomass (LT) and its hydroalcoholic extract (ELT).

FTIR spectroscopy was employed to compare the functional group composition of the raw *L. tridentata* biomass (LT) and the corresponding hydroalcoholic extract (ELT). The spectra obtained for both materials are presented in Figure 2. Both LT and ELT exhibited a broad absorption band centered at 3334.92 and 3272.34 cm^−1^, respectively, which is commonly attributed to O–H stretching vibrations associated with hydroxyl-containing compounds. The presence of these bands suggests the occurrence of oxygenated organic constituents in both samples. Absorption bands observed at 2918.29 cm^−1^ (LT) and 2925.51 cm^−1^ (ELT) were assigned to aliphatic C–H stretching vibrations, indicating the presence of organic structures containing methylene and methyl groups. Additional differences were observed in the fingerprint region.

The ELT spectrum exhibited bands at 1601.68, 1513.27, and 1261.65 cm^−1^, which are commonly associated with aromatic skeletal vibrations and C–O stretching modes of oxygenated aromatic compounds. In contrast, LT exhibited a less pronounced band at 1604.77 cm^−1^, attributed to aromatic C=C vibrations. Furthermore, absorption bands detected at 1701.24 cm^−1^ (ELT) and 1025.92 cm^−1^ (ELT) together with the corresponding band at 1022.27 cm^−1^ (LT) were associated with carbonyl- and oxygen-containing functional groups. Overall, the FTIR results revealed differences between the raw biomass and the hydroalcoholic extract, indicating that the extraction process selectively recovered organic constituents containing hydroxylated, aromatic, and oxygenated functional groups.

#### 2.2.3. Nuclear Magnetic Resonance Analysis of the Hydroalcoholic Extract

**Figure 3:**
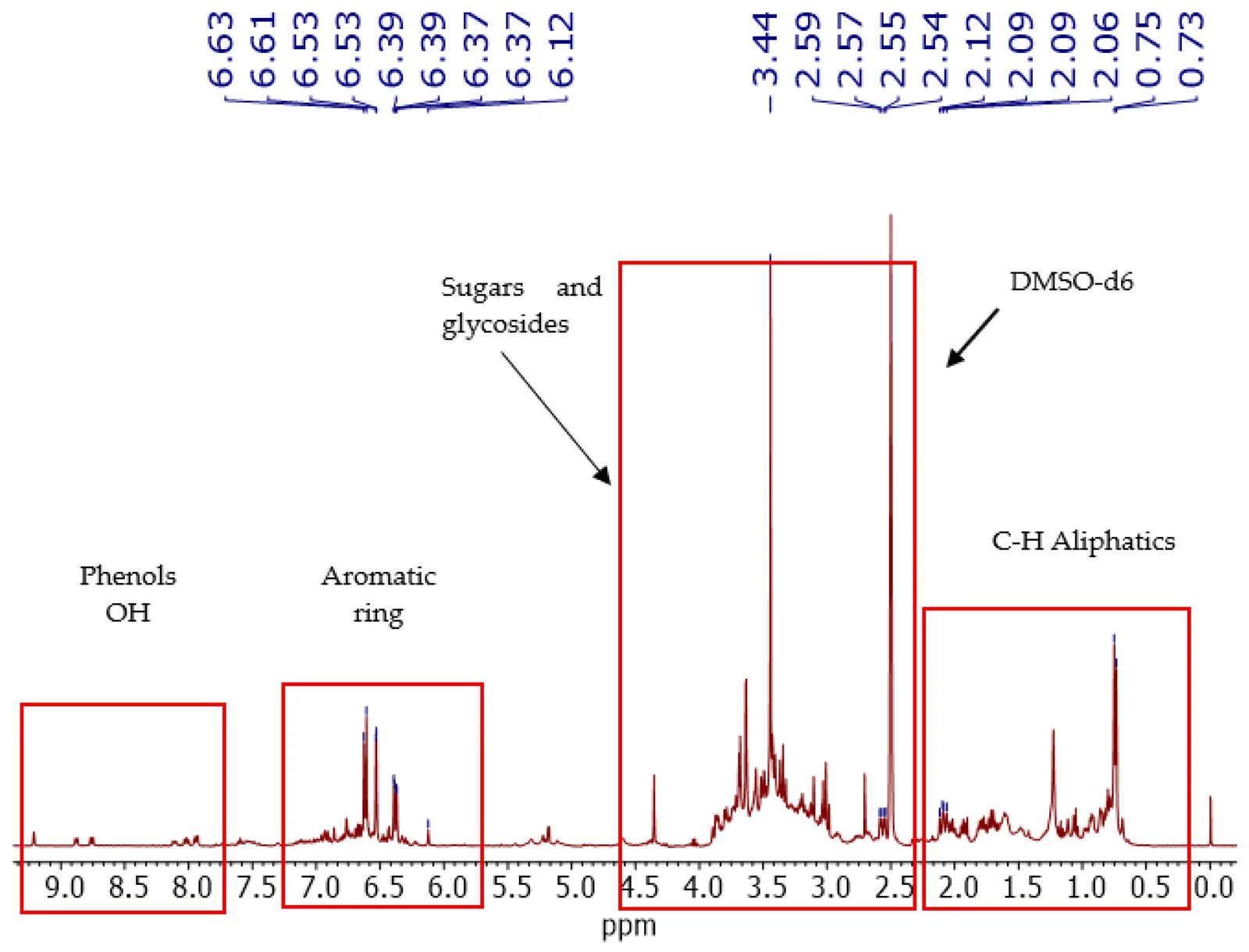
^1^H-NMR of extraction for *L. tridentata* (ELT).

The chemical composition of the ELT sample was further investigated by ^1^H and ^13^C NMR spectroscopy. Rather than targeting the identification of a single compound, NMR analyses were employed to obtain a global spectroscopic fingerprint of the extract and to evaluate the presence of characteristic structural motifs commonly reported for *L. tridentata* metabolites. The ^1^H NMR spectrum (Figure 3) exhibited signals in the aromatic region between approximately 6.3 and 6.7 ppm, indicating the presence of aromatic proton environments associated with phenolic constituents. Additional resonances observed in the aliphatic region were assigned to methyl and methylene groups belonging to structurally diverse organic compounds present in the extract.

The corresponding ^13^C NMR spectrum revealed signals attributable to aromatic carbons, oxygenated aromatic carbons, methylene carbons, and methyl groups. The experimental chemical shifts showed good agreement with values reported in the literature for phenolic and lignan-type constituents previously described in *L. tridentata* extracts, signals consistent with NDGA-like lignan structures (Table 2).

Overall, the NMR results support the presence of a chemically complex mixture enriched in aromatic and oxygenated organic compounds, consistent with the hydroalcoholic extraction of phenolic and other complex phytochemical constituents from *L. tridentata*.

#### 2.2.4. Gas Chromatography-Mass Spectroscopy (GC-MS)

GC–MS analysis was performed to obtain complementary information regarding the chemical composition of the hydroalcoholic extract of *L. tridentata* (ELT). The chromatographic profile revealed nine major constituents, belonging to different classes of phytochemicals, including fatty acid esters, terpenoid derivatives, phenolic compounds, and phytosterols (Table 3).

Among the detected constituents, fatty acid derivatives represented the predominant fraction of the extract, accounting for approximately 64.57% of the total chromatographic area. The most abundant compounds corresponded to 9,12,15-octadecatrienoic acid ethyl ester (28.72%), hexadecanoic acid ethyl ester (23.41%), and 9,12-octadecadienoic acid methyl ester (10.32%). Together, these three compounds represented 62.45% of the chromatographic profile, while the remaining percentage was attributable to other minor fatty acid derivatives. Terpenoid compounds, comprising phytol (15.29%), γ-Sitosterol (8.75%), 2-Naphthalenemethanol derivative (1.78%), and isophytol (0.82%), represented approximately 26.64% of the detected chromatographic profile. Additionally, a phenolic constituent identified as 2-propenoic acid, 3-(4-hydroxy-3-methoxyphenyl)- was detected with a relative area of 8.72%. These results indicate that the hydroalcoholic extraction procedure recovered a chemically diverse mixture of organic compounds containing aromatic, oxygenated, and aliphatic structures. The GC–MS results are consistent with the functional groups identified by FTIR analysis and with the structural features suggested by the NMR spectra, confirming the presence of complex organic constituents within ELT.

### 2.3. Experimental Procedure for the Solar-Driven Transformation of AELT

#### 2.3.1. UV–Vis Spectral Characteristics of AELT

The UV–Vis absorption spectrum of the AELT was monitored (at −30 min). A characteristic absorption band centered at approximately 280 nm was observed, indicating the presence of UV-active organic constituents in the extract. The absorption in this spectral region is commonly associated with electronic transitions involving π-electron systems and nonbonding electrons present in aromatic structures and oxygenated organic compounds. The occurrence of this absorption band is consistent with the presence of phenolic and aromatic constituents previously suggested by the FTIR and NMR analyses of the hydroalcoholic extract.

Because the absorption maximum remained well defined and exhibited high sensitivity to concentration changes, the absorbance at 280 nm was selected as the analytical response for monitoring the transformation of aromatic chromophores and estimating the concentration of AELT during the kinetic analyses. It is important to note, however, that monitoring absorbance at this wavelength specifically tracks the primary transformation and removal of the core aromatic and phenolic chromophores in the extract.

#### 2.3.2. Solar Irradiance and Temperature

**Figure 4:**
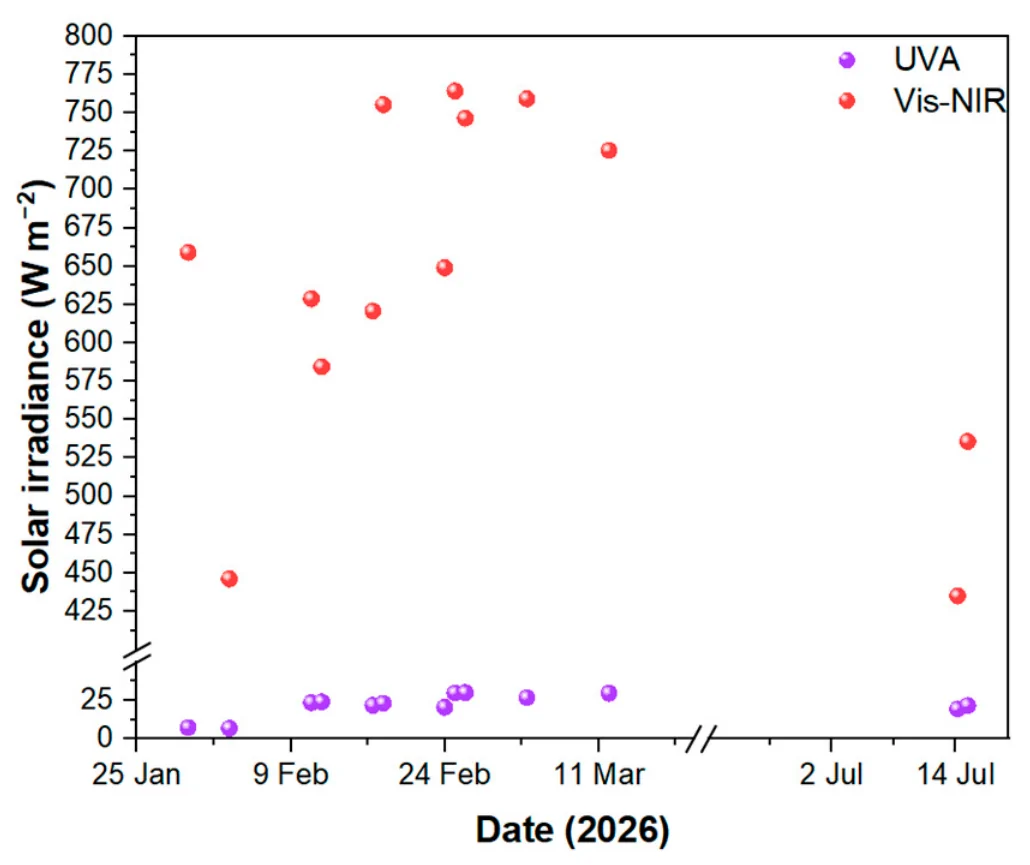
Daily variation in natural solar irradiance: Vis–NIR (red points) and UV-A (purple points) regions measured in Chihuahua, México, during the photocatalytic evaluation period (January–March and July 2026)**.**

The daily variation in natural solar irradiance and ambient temperature recorded during the experimental period is presented in Figure 4. The measured Vis–NIR irradiance (400–1050 nm) ranged from 466 to 764 W m^−2^, with an average value of 663 ± 124 W m^−2^, whereas the UV-A component (315–400 nm) varied between 20.51 and 30.08 W m^−2^, with a mean irradiance of 25 ± 4 W m^−2^. During the experiments, the ambient temperature ranged from 18.0 to 34.2 °C, with an average temperature of 28.7 ± 5.22 °C. These variations in solar irradiance and temperature reflect the natural environmental conditions under which the photocatalytic experiments were conducted.

#### 2.3.3. UV–Vis Spectral Evolution During Solar Transformation

**Figure 5:**
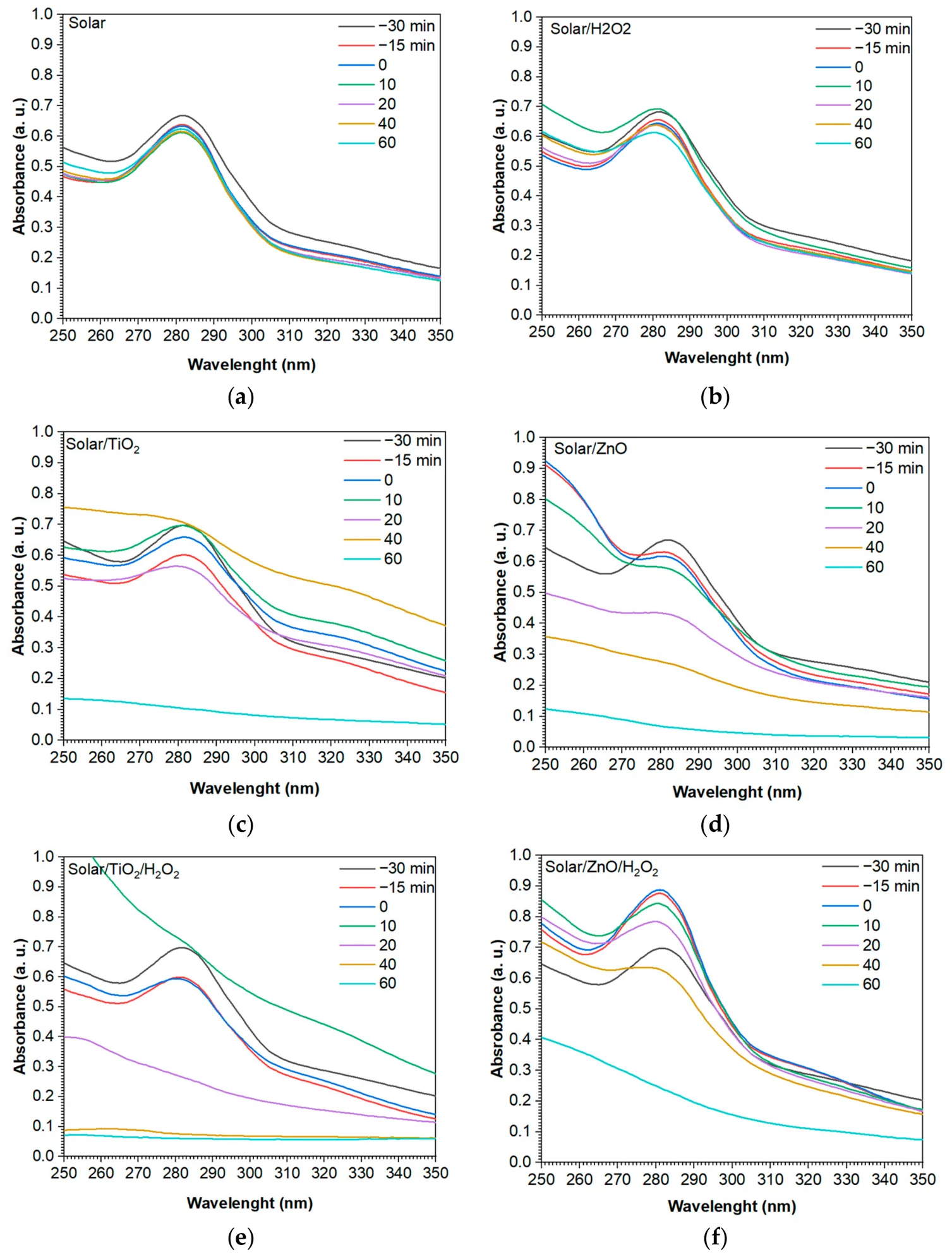
UV–Vis absorption spectra during dark adsorption (−30 to 0 min) and solar-driven chromophore removal (0 to 60 min) of AELT for the evaluated systems: (**a**) Solar, (**b**) Solar/H_2_O_2_, (**c**) Solar/TiO_2_, (**d**) Solar/ZnO, (**e**) Solar/TiO_2_/H_2_O_2_, and (**f**) Solar/ZnO/H_2_O_2_.

The evolution of the UV–Vis absorption spectra of AELT during the solar-driven chromophore removal experiments is presented in Figure 5. Spectra were recorded after the dark adsorption–desorption equilibration period (−30 and −15 min), immediately before solar irradiation (0 min), and after 10, 20, 40, and 60 min of exposure. For all evaluated systems, the characteristic absorption band centered near 280 nm remained identifiable throughout the experiments, although absorbance decreased progressively depending on the treatment applied. This spectral region was selected as the analytical indicator of AELT chromophore removal because it exhibited the highest absorbance and was associated with the aromatic and oxygenated organic constituents present in the extract.

The Solar and Solar/H_2_O_2_ systems exhibited only minor spectral variations during irradiation, indicating limited removal of UV-active compounds under these conditions. In contrast, the photocatalytic systems containing TiO_2_ or ZnO showed a progressive decrease in absorbance across the monitored spectral range, indicating the removal of chromophores from extract constituents under solar irradiation. Among the evaluated treatments, Solar/TiO_2_/H_2_O_2_ and Solar/ZnO produced the most pronounced reductions in absorbance intensity. In particular, the Solar/TiO_2_/H_2_O_2_ system exhibited a substantial decrease in the absorption band after 20 min of irradiation, while the spectra recorded at 40 and 60 min showed markedly lower absorbance values. Similar behavior was observed for Solar/ZnO, which also promoted a significant attenuation of the characteristic absorption band. Overall, the progressive reduction in absorption signals suggests the transformation and removal of UV-active organic compounds present in AELT, with the extent of spectral changes strongly dependent on the chromophore-removal system employed.

#### 2.3.4. Adsorption and Chromophore Removal Efficiencies

**Figure 6:**
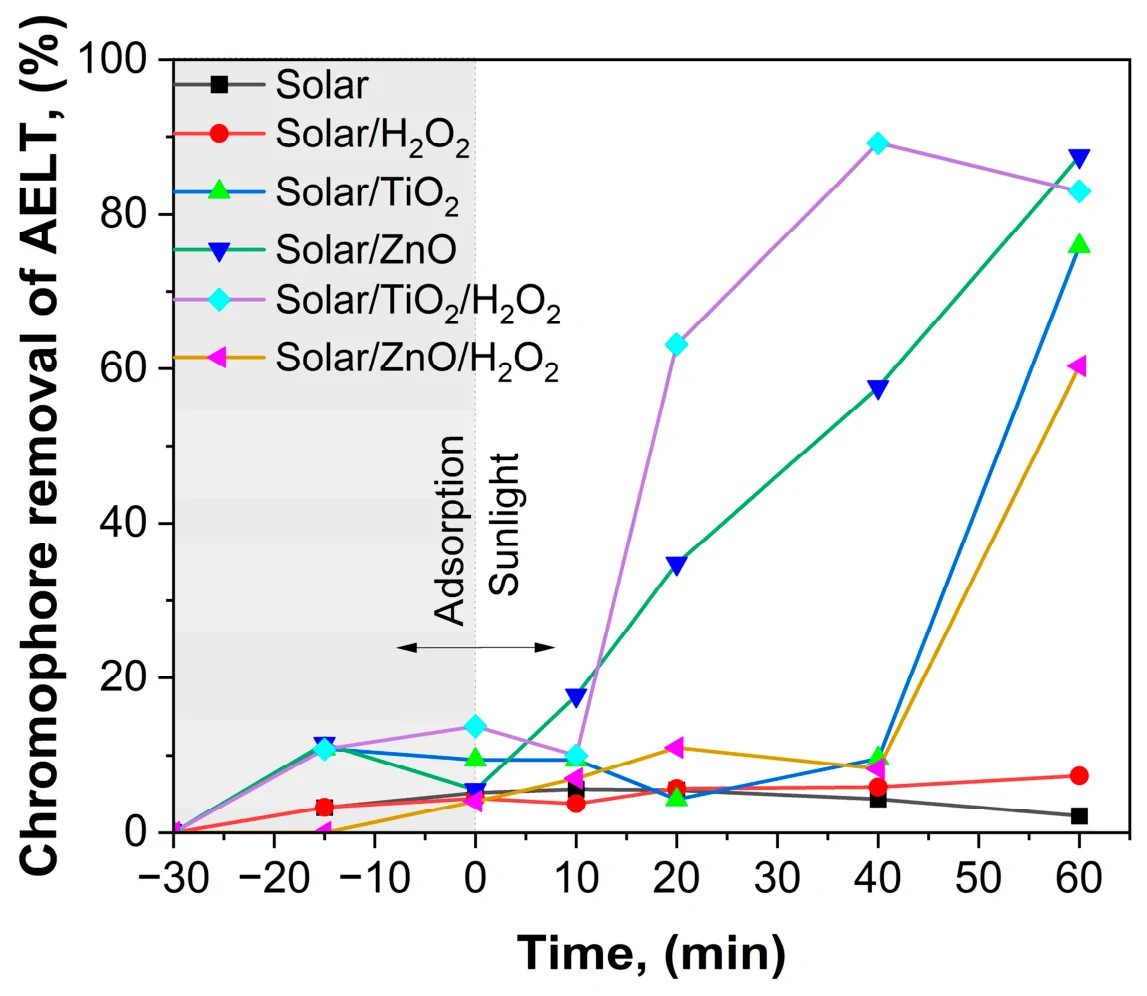
Temporal evolution of the adsorption and chromophore removal efficiencies of AELT under the evaluated solar-driven oxidation systems.

The corresponding adsorption and chromophore removal profiles are presented in Figure 6, using the calibration curve provided in Figure S1 (Supplementary Materials). Also, the final adsorption and chromophore removal efficiencies after 30 min in the dark and 60 min of solar irradiation, respectively, are summarized in Table 4.

The dark adsorption results summarized in Table 4 indicate that the removal of AELT due to physical interactions with the catalyst surface was relatively low across all evaluated systems, with values ranging from 4.12 ± 1.94% to 13.76 ± 2.34%. The highest adsorption was observed for the Solar/TiO_2_/H_2_O_2_ system (13.76 ± 2.34%), while the Solar/ZnO treatment—which achieved the highest overall photocatalytic performance—showed an initial removal of only 5.51 ± 0.72%. These values, obtained after the 30-min dark equilibration period, confirm that only a minor fraction of the extract constituents was removed from the aqueous medium prior to the onset of solar irradiation.

The chromophore removal efficiencies summarized in Table 4 reveal substantial differences among the evaluated solar-driven oxidation systems. The highest removal efficiencies after 60 min of irradiation were achieved with heterogeneous photocatalytic treatments employing semiconductor catalysts. Solar/ZnO achieved the highest chromophore removal efficiency (89.43 ± 0.69%), followed by Solar/TiO_2_/H_2_O_2_ (84.34 ± 6.86%) and Solar/TiO_2_ (71.36 ± 1.93%). In contrast, direct solar photolysis and the Solar/H_2_O_2_ system exhibited limited chromophore removal efficiencies, with values below 10%, indicating that neither solar irradiation alone nor the addition of H_2_O_2_ in the absence of a semiconductor catalyst was sufficient to promote efficient AELT chromophore removal under the evaluated conditions. Although the combined TiO_2_/H_2_O_2_ system exhibited a chromophore removal efficiency comparable to that obtained with ZnO alone, the latter achieved the highest removal efficiency with considerably lower experimental variability, suggesting a more reproducible photocatalytic performance under the solar irradiation conditions studied.

The Kolmogorov–Smirnov test confirmed that the experimental data satisfied the normality assumption (Table S1, Supplementary Materials), supporting the application of parametric statistical analysis. One-way ANOVA revealed statistically significant differences among the six chromophore removal systems (F = 361.09, *p* < 0.0001), indicating that the chromophore removal treatment significantly affected AELT removal efficiency (Table 5).

Post hoc comparison using the least significant difference (LSD) test showed that most treatments differed significantly from one another (*p* < 0.05). The only comparisons that did not present statistically significant differences were Solar versus Solar/H_2_O_2_, Solar/ZnO versus Solar/TiO_2_/H_2_O_2_, and Solar/TiO_2_ versus Solar/ZnO/H_2_O_2_ (Table 6).

These results indicate that incorporating semiconductor photocatalysts resulted in a marked improvement in chromophore removal performance compared with photolysis or H_2_O_2_ alone. Furthermore, the statistically equivalent performance observed between Solar/ZnO and Solar/TiO_2_/H_2_O_2_ suggests that ZnO alone could be capable of achieving chromophore removal efficiencies comparable to those obtained with the combined TiO_2_/H_2_O_2_ oxidation system under the evaluated solar irradiation conditions.

#### 2.3.5. Kinetic Analysis

**Figure 7:**
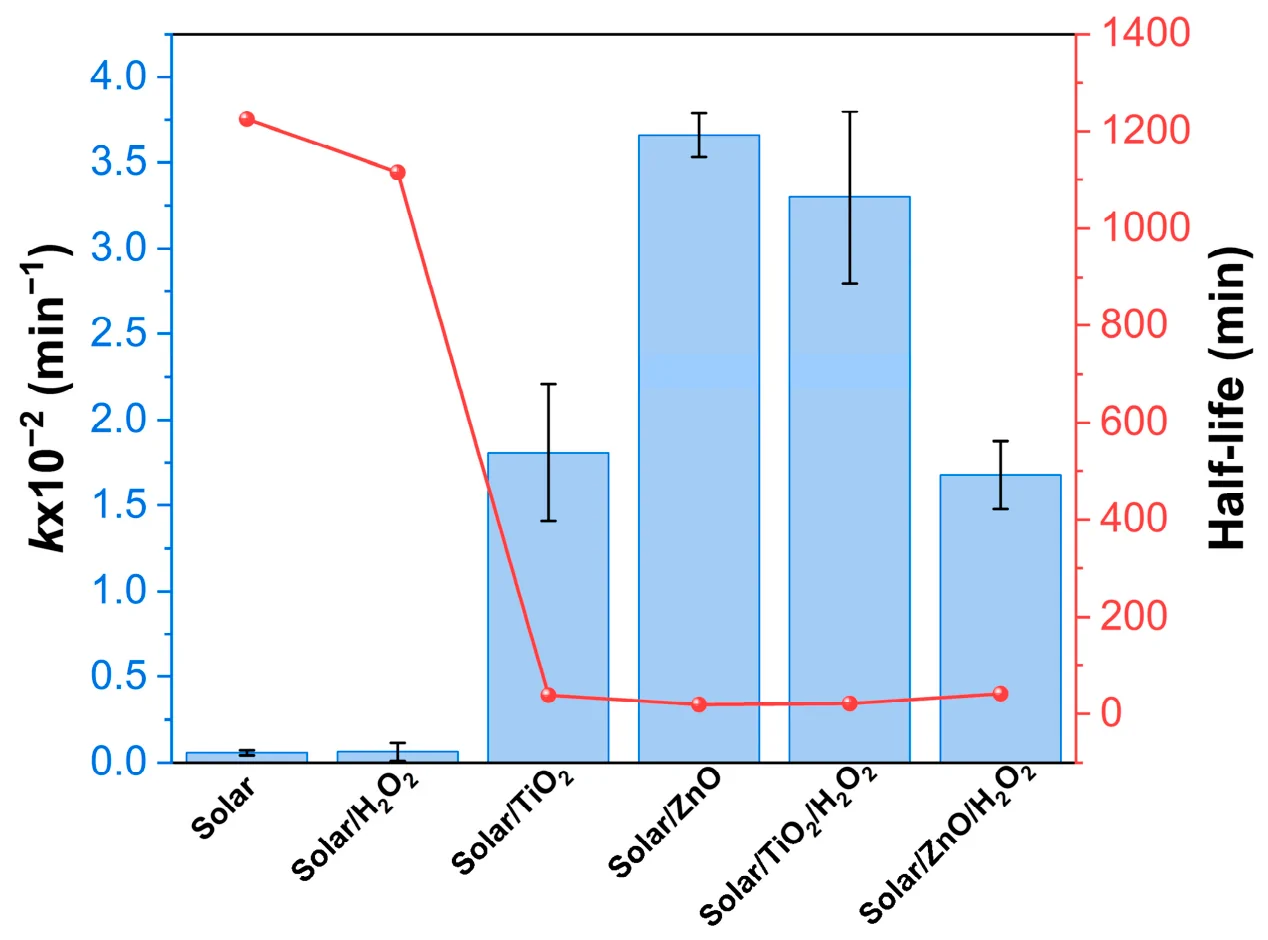
Pseudo-first-order kinetic plots and kinetic parameters (*k* and *t*_1/2_) for the solar-driven transformation of AELT under the evaluated oxidation systems.

The corresponding kinetic plots, such as the apparent rate constants (kapp) and half-life (*t*_1/2_), are presented in Figure 7.

Marked differences in the chromophore removal kinetics were observed among the evaluated treatments. The highest *k*_app_ values were obtained for the Solar/ZnO and Solar/TiO_2_/H_2_O_2_ systems, with values of (3.67 ± 1.31) × 10^−2^ and (3.31 ± 0.53) × 10^−2^ min^−1^, respectively, indicating faster chromophore removal of AELT under these experimental conditions. In contrast, direct solar photolysis and the Solar/H_2_O_2_ treatment exhibited rate constants below 1 × 10^−3^ min^−1^, indicating that solar irradiation alone, or combined with H_2_O^2^, resulted in substantially slower chromophore removal. The relatively low standard deviations obtained for most treatments indicate good experimental reproducibility.

The estimated *t*_1/2_ values were consistent with the corresponding chromophore removal rate constants. The shortest half-lives were obtained for the Solar/ZnO (18.92 ± 0.14 min) and Solar/TiO_2_/H_2_O_2_ (21.02 ± 0.93 min) systems, whereas direct solar photolysis and Solar/H_2_O_2_ exhibited considerably longer half-lives of 1225.00 ± 0.81 and 1114.83 ± 0.71 min, respectively. The Solar/ZnO/H_2_O_2_ and Solar/TiO_2_ treatments showed intermediate *t*_1/2_ values of 41.36 ± 0.03 and 38.28 ± 0.10 min, respectively. Overall, these kinetic parameters are consistent with the chromophore removal efficiencies reported in Section 2.3.4 and further demonstrate the superior performance of semiconductor-assisted solar oxidation systems in removing the d chromophore from AELT. Because these *t*_1/2_ values substantially exceed the 60-min observation window, they should be regarded as model-derived apparent estimates rather than experimentally observed half-lives.

### 2.4. Organic Matter Oxidation Assessed by COD

**Figure 8:**
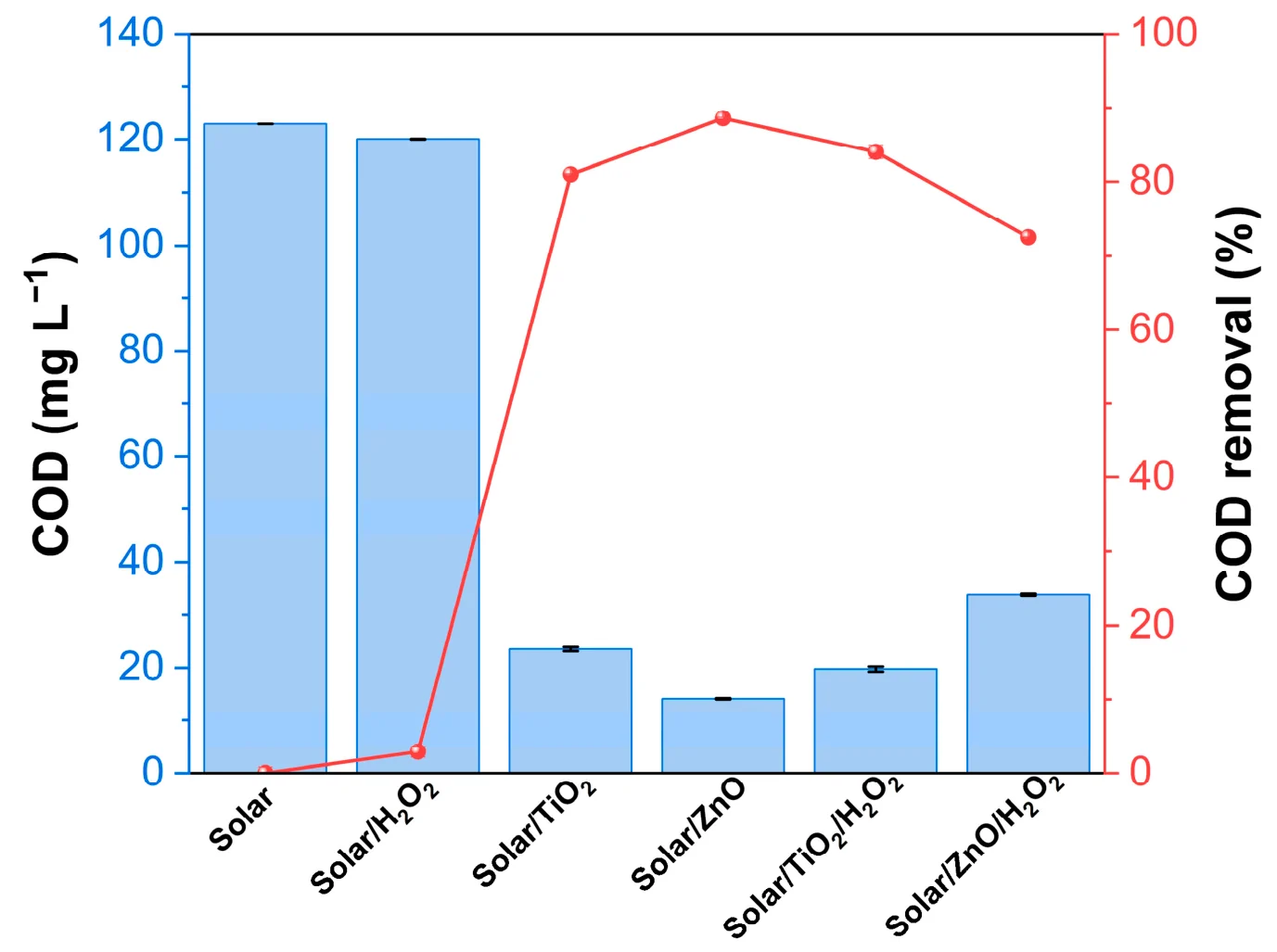
Final COD removal efficiencies and remaining organic load of AELT after 60 min of treatment under the evaluated solar-driven oxidation systems.

The COD values measured after 60 min of solar irradiation are summarized in Figure 8. The initial COD of the AELT solution was 123.7 mg L^−1^. Under direct solar irradiation, the COD remained essentially unchanged (122.9 mg L^−1^), indicating that solar photolysis alone produced no measurable oxidation of the dissolved organic matter. Similarly, the Solar/H_2_O_2_ system showed only a slight decrease in COD to 120.13 ± 0.87 mg L^−1^, corresponding to a removal efficiency of 2.96 ± 0.69%.

In contrast, the photocatalytic systems containing semiconductor catalysts exhibited substantially higher COD removal efficiencies. The Solar/TiO_2_ treatment reduced the COD to 23.47 ± 0.81 mg L^−1^, corresponding to an oxidation efficiency of 81.29 ± 0.67%, whereas the Solar/ZnO system achieved the highest performance, decreasing the COD to 14.22 ± 0.31 mg L^−1^ with a removal efficiency of 88.78 ± 0.18%. Likewise, the combined Solar/TiO_2_/H_2_O_2_ system reached a final COD of 19.38 ± 0.84 mg L^−1^, corresponding to an oxidation efficiency of 83.44 ± 0.75%. The Solar/ZnO/H_2_O_2_ treatment also promoted substantial oxidation of organic matter, achieving a COD removal efficiency of 71.66 ± 0.76%.

## 3. Discussion

### 3.1. Physicochemical Characteristics of the Hydroalcoholic Extract

The substantial extraction yield (26.36%) and the high volatile solid content confirm the efficacy of the Soxhlet procedure in concentrating organic metabolites from *L. tridentata*. This efficiency is attributed to the selective recovery of medium-polarity secondary metabolites facilitated by the ethanol–water solvent system, which effectively shifts the material’s relative chemical composition toward solvent-soluble constituents [17].

The extraction yield of 26.36%, together with the volatile-solid content, indicates substantial recovery of solvent-soluble organic constituents from *L. tridentata* under the applied Soxhlet conditions. The use of an ethanol–water mixture is consistent with previous studies showing that hydroalcoholic solvents can effectively extract phenolic and other secondary metabolites from plant matrices, owing to their intermediate polarity and ability to solubilize compounds of varying polarities [26]. In *L. tridentata*, the solvent composition has been shown to significantly influence the recovery of lignans and flavonoids, further supporting the role of solvent polarity in determining extract composition [27].

TGA/DTG further supports the compositional changes associated with the extraction process, showing a lower ash content in the extract (ELT) than in the raw biomass and a predominant mass-loss event between 200 and 350 °C. This temperature region is commonly associated with the thermal decomposition of hemicellulosic and other oxygenated organic fractions in plant biomass [28]. Because plant-derived matrices contain overlapping contributions from extractives, hemicellulose, cellulose, and lignin, the observed thermal event should not be assigned to a single chemical constituent [29]. When considered together with the FTIR, NMR, and GC-MS characterization, the TGA/DTG profile is consistent with the recovery of solvent-soluble organic constituents from the biomass during Soxhlet extraction [30].

Spectroscopic characterization provided a more detailed molecular description of the extracted constituents. FTIR spectra of ELT displayed intense O–H stretching bands, together with aromatic and carbonyl vibrations, consistent with the presence of oxygenated and aromatic constituents commonly reported in polyphenol- and lignan-containing plant extracts [31,32]. These observations were further complemented by ^1^H and ^13^C NMR spectroscopy, which provided a global molecular fingerprint showing the coexistence of aromatic, oxygenated, and aliphatic chemical environments characteristic of complex *Larrea* matrices [33]. GC-MS analysis further complemented the spectroscopic results by identifying bioactive compound classes, including terpenoids, phytosterols, and fatty acid derivatives, whose occurrence is consistent with the functional groups and chemical environments detected by FTIR and NMR [34].

Collectively, these physicochemical characteristics provide a basis for interpreting the optical behavior of ELT in aqueous solution. The pronounced absorption around 280 nm is consistent with the contribution of aromatic and phenolic constituents, as phenolic compounds commonly exhibit absorption in this spectral region due to their aromatic and conjugated electronic structures [35]. Accordingly, the 280 nm signal was used as an operational indicator to monitor changes in the UV-active chromophoric fraction during the subsequent photocatalytic treatment [36]. This approach is also consistent with previous characterization of *L. tridentata* ethanol–water extracts using chromatographic detection at 280 nm [37].

### 3.2. Solar-Driven Transformation of AELT

The limited removal observed in the absence of irradiation (Figure 6, Table 4) indicates that surface adsorption made only a minor contribution to the overall removal under the experimental conditions. Although TiO_2_ and ZnO can interact with organic molecules through surface adsorption, their primary function in the present systems was photocatalytic oxidation upon irradiation [38]. Establishing the contribution of the dark-equilibration step therefore provides an experimental basis for distinguishing removal associated with surface interactions from that associated with the subsequent light-driven transformation. The substantially greater removal observed after exposure to natural sunlight is consequently consistent with a predominant contribution from irradiation-driven photocatalytic processes rather than adsorption alone [38,39].

The effectiveness of natural sunlight as an excitation source should be evaluated in terms of both irradiance and photon energy [40]. Although the Vis–NIR region represented the major fraction of the measured solar irradiance, photons within this spectral range generally do not provide sufficient energy to promote the intrinsic valence-band-to-conduction-band transition of pristine TiO_2_ and ZnO, whose wide band gaps are typically around 3.0–3.2 eV for TiO_2_ and approximately 3.2–3.4 eV for ZnO [41,42]. In contrast, the shorter-wavelength portion of the UV-A region provides photons with sufficient energy to overcome these band gaps and generate photogenerated electron–hole pairs [43,44]. Consequently, the relatively modest UV-A irradiance measured during the experiments may have been more directly relevant to semiconductor activation than the substantially higher Vis–NIR irradiance. This interpretation is consistent with previous results from our research group obtained for Rhodamine B degradation using ZnO under natural sunlight. In that study, effective photocatalytic activity was observed under a mean UV-A irradiance of 25.18 ± 4.73 W m^−2^, with a much higher Vis–NIR irradiance of 730.73 ± 44.9 W m^−2^, and the ZnO exhibited a band gap of approximately 3.18 eV [23]. Although the experiments were performed during a different period of the year, the comparable UV-A irradiance provides experimental support for the relevance of this spectral region to ZnO activation under natural-solar conditions. Nevertheless, the present experiments were not designed to isolate the contribution of individual spectral regions; therefore, the relative contribution of UV-A to the overall process should be regarded as a mechanistic interpretation rather than as a directly quantified contribution.

The enhanced transformation observed in the semiconductor-containing systems is consistent with the ability of TiO_2_ and ZnO to promote light-driven interfacial redox reactions through photogenerated charge carriers [43,45]. This behavior is particularly relevant for AELT because its molecular characterization indicates a chemically heterogeneous matrix containing aromatic and oxygenated functionalities that may undergo oxidative transformation. Accordingly, the photocatalytic response should be interpreted as the collective transformation of multiple classes of phytochemical constituents rather than the degradation of a single target compound. Because reactive species were not directly identified in the present study, the involvement of specific species, such as hydroxyl radicals, remains a plausible mechanistic interpretation rather than an experimentally demonstrated process [45].

The contrasting effect of H_2_O_2_ further indicates that peroxide addition cannot be considered universally beneficial for semiconductor-assisted solar oxidation. Its positive effect in the TiO_2_ system is consistent with the established role of H_2_O_2_ as an electron acceptor and additional source of oxidizing species [40], which can promote charge utilization and enhance oxidative reactions under suitable conditions [46]. Importantly, the effect of H_2_O_2_ is concentration-dependent, and previous studies have reported synergistic effects at appropriate concentrations followed by inhibition when the peroxide concentration becomes excessive [47]. In the present ZnO system, the lower performance observed after H_2_O_2_ addition suggests that the employed concentration did not provide a net oxidative benefit. Competition for reactive species and radical-scavenging reactions have been proposed as possible explanations for inhibitory effects at elevated H_2_O_2_ concentrations [46]. Surface interactions between peroxide and ZnO could also contribute to the observed response, although this possibility was not directly investigated in the present study. Under the conditions evaluated, the high activity obtained with ZnO in the absence of external H_2_O_2_ indicates that effective solar-driven transformation of AELT can be achieved without peroxide addition, whereas the TiO_2_ system benefited from peroxide supplementation. This contrast highlights the importance of optimizing the semiconductor–oxidant combination rather than examining whether additional H_2_O_2_ will universally improve photocatalytic performance.

The decrease in the absorption band around 280 nm provides evidence of the progressive transformation of aromatic and phenolic chromophores present in AELT [36]. For a complex extract, however, changes in this spectral region cannot be equated directly with complete degradation of the organic matrix because different constituents may contribute to the same absorption band [48]. The COD measurements consequently provide a complementary measure of the overall oxidizable organic fraction [49]. The consistency between the spectral and COD trends indicates that the most effective solar-driven systems promoted transformations beyond isolated changes in UV-absorbing chromophores, resulting in a substantial decrease in the overall oxidizable organic load. Nevertheless, COD removal should not be interpreted as evidence of complete mineralization, since COD reduction does not distinguish between the disappearance of parent compounds and the formation of partially oxidized transformation products [50].

## 4. Materials and Methods

### 4.1. Plant Material and Chemicals

The collection and preparation of *L. tridentata* vegetative material followed a standardized procedure previously reported for this species in the arid regions of northern Mexico [51]. Samples were collected during the week of 12 April 2024, from the surroundings of the Francisco I. Madero dam (28°7′58″ N, 105°40′12″ W). In accordance with established protocols for the study of creosote bush, the material was identified based on its distinctive morphological features and environmental context. The biomass was subsequently air-dried at room temperature, ground to a fine powder (MAQNOVA, Lençóis Paulista, Brazil, model MPM-150), and stored in a completely closed, dark container to preserve its chemical integrity prior to extraction.

Commercial titanium dioxide (TiO_2_, Degussa, Essen, Germany, P25, ≈21 nm) and zinc oxide (ZnO, Sigma-Aldrich, St. Louis, MO, USA, <50 nm) were employed as photocatalysts in the solar-driven advanced oxidation experiments. Hydrogen peroxide (H_2_O_2_, Sigma-Aldrich) was used as an oxidizing agent. Ethanol (99.8%) and triple-distilled water (J. T. Baker) were used to prepare all aqueous solutions and dispersions.

### 4.2. Biomass Preparation and Hydroalcoholic Extraction

The plant material was selected based on its characteristic morphology, commonly associated with *L. tridentata* in arid regions of northern Mexico.

The collected biomass was air-dried at room temperature. After drying, the material was cut into segments of approximately 5 cm with gardening scissors and subsequently ground in a pulverizing mill (MAQNOVA, model MPM-150) to obtain a homogeneous powder (0.149 mm) suitable for extraction.

The powdered biomass was subjected to Soxhlet extraction following a procedure adapted from previously reported methodologies [52]. Briefly, 50.0 g samples of dried and powdered biomass were subjected to Soxhlet extraction using 500 mL of an ethanol:water mixture (70:30, *v*/*v*) to ensure a solid-to-liquid ratio of 1:10. After 15 h, the resulting liquid was concentrated to obtain the crude ELT.

After extraction, the solvent was removed using a rotary evaporator (BUCHI R-210, Labortechnik AG, Flawil, Switzerland) at 45 °C under reduced pressure, yielding a concentrated crude extract with a final vacuum of 30 mbar. All extraction procedures were performed in triplicate to ensure reproducibility.

The extraction yield was calculated according to Equation (1):

*Yield* (%) = (*W_f_*/*W_i_*) × 100, (1)

where *W_f_* is the final weight of the recovered crude extract (g), and *W_i_* is the initial weight of the dried biomass (g).

For clarity, the samples analyzed throughout this study were designated as follows: LT corresponds to the raw *L. tridentata* biomass after drying and milling; ELT corresponds to the hydroalcoholic extract obtained by Soxhlet extraction using an ethanol mixture (70:30, *v*/*v*); and AELT corresponds to aqueous dispersions prepared from ELT using triple-distilled water and subsequently employed in the solar-driven chromophore removal experiments.

### 4.3. Physicochemical Characterization of L. tridentata Biomass and Hydroalcoholic Extract

The raw *L. tridentata* biomass (LT) and the corresponding hydroalcoholic extract (ELT) were characterized using complementary analytical techniques to evaluate their thermal behavior, functional groups, and chemical composition. LT and ELT were analyzed by thermogravimetric analysis (TGA/DTG) and Fourier-transform infrared spectroscopy (FTIR). The chemical composition of ELT was further investigated by ^1^H/^13^C nuclear magnetic resonance (NMR) spectroscopy and gas chromatography–mass spectrometry (GC–MS). The aqueous hydroalcoholic extract (AELT) was characterized by UV–Vis spectroscopy prior to the chromophore removal experiments and subsequently monitored during the solar-driven chromophore removal assays.

TGA/DTG were performed to determine moisture content, volatile solids, ash content, and thermal stability. Thermal analyses were carried out using a simultaneous DSC-TGA instrument (STD Q600, New Castle, DE, USA) under an argon/air atmosphere with a flow rate of 50 mL min^−1^. Approximately 10 mg of sample was placed in alumina crucibles and heated from 30 to 800 °C at a heating rate of 10 °C min^−1^. The corresponding DTG curves were obtained from the TGA profiles; the moisture content was determined by heating the sample to 107 °C until a constant weight was reached. Subsequently, volatile matter was evaluated by ramping the temperature at 10 °C min^−1^ up to 800 °C, then holding isothermally for 7 min under the same atmosphere to ensure pyrolytic conditions. Finally, the ash content was determined after complete combustion of the residual fixed carbon until baseline stabilization.

Functional groups present in the biomass and hydroalcoholic extract were identified by FTIR spectroscopy of each sample, which was recorded in the mid-infrared (MIR) region using a Shimadzu, Kyoto, Japan, IRAffinity-1S spectrophotometer equipped with an attenuated total reflectance (ATR) accessory. FTIR analyses were carried out with 30 scans at a resolution of 4 cm^−1^ over the 4000–400 cm^−1^ frequency range to evaluate differences in functional group composition between the raw biomass and the corresponding hydroalcoholic extract.

NMR spectroscopy was employed to structurally characterize ELT. Spectra were recorded using a Bruker ASCEND 400 spectrometer (Karlsruhe, Germany) operating at 400 MHz for ^1^H and 100 MHz for ^13^C at 298 K. A total of 32 scans were acquired using a relaxation delay (D1) of 10 s.

For sample preparation, 20 mg of ELT was dissolved in deuterated dimethyl sulfoxide (DMSO-*d*_6_) and transferred into 5 mm NMR tubes. Chemical shifts (δ) were reported in ppm and referenced to the residual solvent signals at 2.50 ppm for ^1^H and 39.43 ppm for ^13^C. Signal multiplicities were assigned according to standard conventions as s (singlet), d (doublet), t (triplet), and q (quartet). Raw data processing, Fourier transform, phasing, and baseline corrections were performed using MestReNova software version 12.0.2-20910. Structural assignments of key peaks were subsequently performed by cross-referencing experimental chemical shifts and multiplicities with established literature standards and the spectral database (SDBS) for the characteristic lignan frameworks of *L. tridentata* [53,54].

For the GC-MS, the ELT sample was dissolved in dichloromethane, and the resulting extract was analyzed by gas chromatography–mass spectrometry (GC–MS). Analyses were performed on a Shimadzu Kyoto, Japan, GCMS-QP2010 SE fitted with an Rxi-5Sil MS capillary column (30.0 m × 0.25 mm i.d., 0.25 µm film thickness). Helium was used as the carrier gas with linear velocity control. The injector (INJ1) was operated in split mode (split ratio 5.0) at 320 °C. The MS was operated in scan mode with the ion source and transfer/interface temperatures set to 200 °C. The column oven was programmed as described below (total program time 32.0 min). Injection volume and injection mode details (split-flow specifics) were as used on the instrument.

Instrumental conditions:

Instrument: Shimadzu GCMS-QP2010 SE.

Column: Rxi-5Sil MS, 30.0 m × 0.25 mm i.d., 0.25 µm film thickness.

Carrier gas: Helium; flow control: linear velocity; linear velocity = 36.5 cm s^−1^.

Column flow: 1.00 mL min^−1^; total flow: 9.0 mL min^−1^; inlet (head) pressure ≈ 57.5 kPa; purge flow = 3.0 mL min^−1^.

Injector: INJ1, split mode, split ratio 5.0, injector temperature 320 °C.

MS mode: Scan; ion source temperature = 200 °C; transfer/interface temperature = 200 °C.

Column oven program (as displayed): initial temperature 60 °C (hold 2.0 min); ramp at ~10 °C min^−1^ to ~310–320 °C with a final hold (program total time = 32.0 min).

### 4.4. Solar-Driven Photocatalytic Evaluation

AELT dispersions were prepared at an initial concentration of 75 mg L^−1^ in tri-distilled water containing 75 mg of ELT extract, then stirred gently until fully dissolved. After this process, the solution was transferred to a volumetric flask, brought to a final volume of 1 L, and stored in the refrigerator. These dispersions corresponded to the water-dispersible fraction of the hydroalcoholic extract and were subsequently used in the solar-driven chromophore removal experiments.

Photocatalytic experiments were carried out under natural solar irradiation in Chihuahua, Mexico (28°41′37″ N, 106°05′23″ W), between 11:00 a.m. and 3:00 p.m. local time, corresponding to the period of highest daily solar intensity. Solar irradiance was monitored using a radiometer (Delta Ohm HD 2302.0, Padua, Italy) equipped with LP 471 UVA (315–400 nm) and LP 471 RAD (400–1050 nm) sensors, recording irradiance values in W m^−2^ throughout the experiments.

The evaluated systems included: (i) solar photolysis (solar), (ii) solar/H_2_O_2_, (iii) solar/TiO_2_, (iv) solar/ZnO, (v) solar/TiO_2_/H_2_O_2_, and (vi) solar/ZnO/H_2_O_2_. To ensure the reliability of the results, appropriate blanks and controls were integrated into the experimental design. Triple-distilled water was used as the analytical blank for both UV–Vis and COD measurements to ensure baseline stability. Furthermore, direct solar photolysis (Solar) and the Solar/H_2_O_2_ system (in the absence of catalysts) were established as experimental controls to assess AELT’s susceptibility to direct phototransformation and homogeneous oxidation, respectively. These controls provided the necessary reference to quantify the marked enhancement in chromophore removal efficiency achieved by the heterogeneous photocatalytic systems (TiO_2_ and ZnO). For heterogeneous photocatalytic experiments, 10 mg of TiO_2_ or ZnO was added to the reaction medium under continuous magnetic stirring. In oxidation systems containing H_2_O_2_, 1.97 mM of H_2_O_2_ (30% w/w in H_2_O, Sigma-Aldrich) was added at the beginning of the experiment.

**Figure 9:**
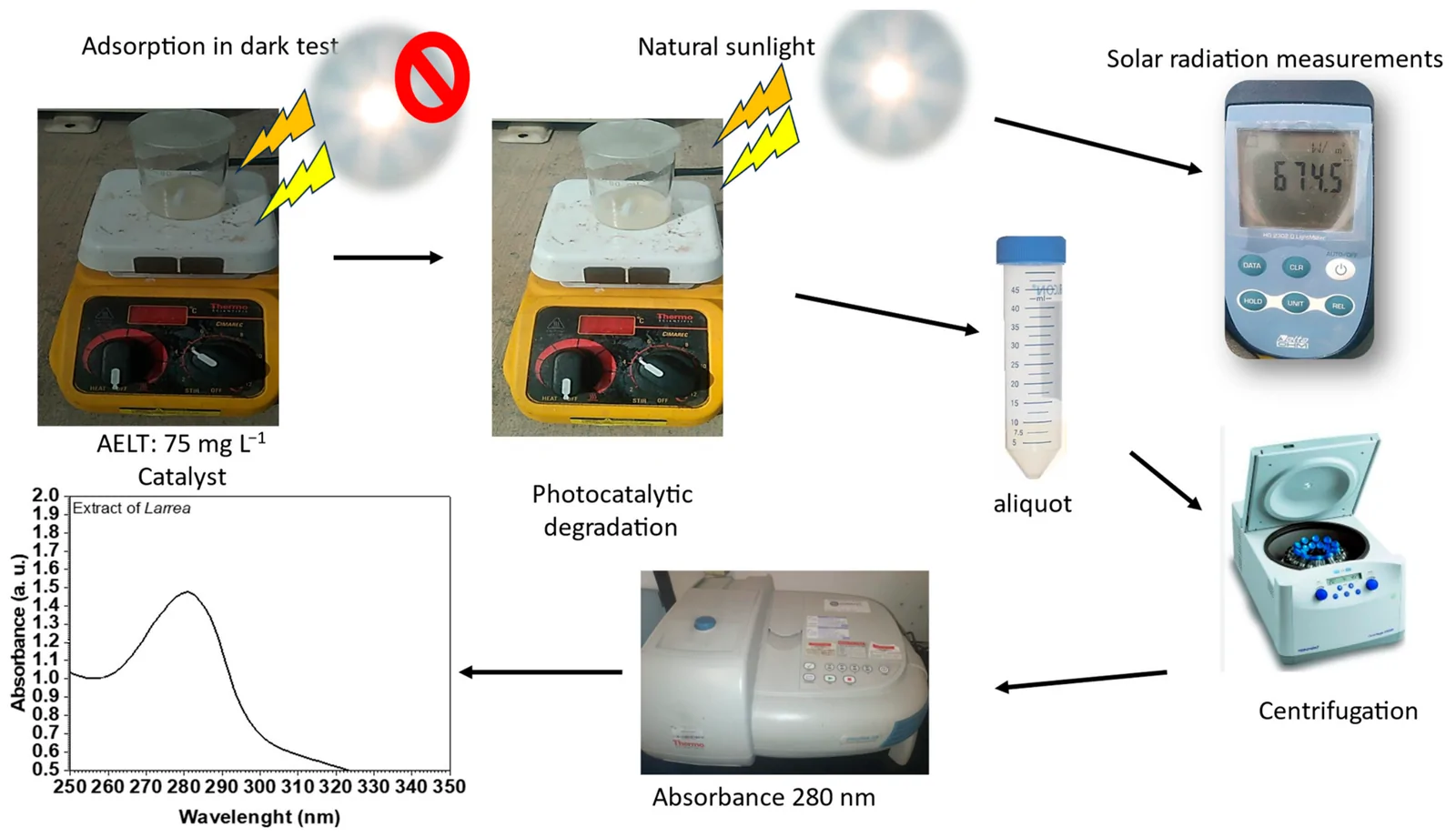
Experimental setup for the solar-driven chromophore removal of AELT.

For all photocatalytic assays, a catalyst dosage of 10 mg was maintained in a total working volume of 50 mL, corresponding to a concentration of 0.2 g L^−1^. The commercial semiconductors employed were TiO_2_ (Degussa P25), a well-known nanoparticulate standard with a mean particle size of ~21 nm, and ZnO (Sigma-Aldrich). Experiments were conducted in cylindrical glass reactors with a liquid column height (optical path length) of approximately 3.5 cm. This parameter is crucial, as it dictates the progressive attenuation of solar radiation through the suspension, which can decrease photon absorption efficiency with increasing path length, a phenomenon thoroughly discussed in recent literature [21,25]. To prevent evaporation of the aqueous medium during solar exposure, the reaction beakers were covered/sealed with Parafilm throughout the irradiation period. This ensured that the observed changes in AELT concentration were exclusively due to chromophore removal. The experimental setup used for the solar-driven chromophore removal assays is presented in Figure 9.

Prior to solar irradiation, all suspensions were magnetically stirred in the dark for 30 min to establish adsorption–desorption equilibrium between the extract constituents and the photocatalyst surface. Aliquots were collected at −30 and −15 min to monitor changes during this dark equilibration period.

The chromophore removal experiments were conducted for 60 min, during which 3 mL aliquots were collected at 0, 10, 20, 40, and 60 min. Samples were centrifuged at 9660× *g* relative centrifugal force (RCF) for 5 min to remove suspended catalyst particles. The total volume removed (18 mL) represents 36% of the initial reaction medium; this volume was strictly necessary to meet the requirements of the standard 1 cm path-length quartz cuvettes used for UV–Vis analysis. To ensure the kinetic integrity of the process and avoid cross-contamination, the collected samples were not returned to the reactor. All aliquots were stored in the dark and analyzed sequentially at the end of the 60 min irradiation period to ensure analytical consistency and minimize instrumental drift.

UV–Vis measurements were performed using a Thermo Scientific Evolution 220 spectrophotometer over the wavelength range of 190–800 nm. The instrument was operated with a spectral bandwidth of 1.0 nm and a scan speed of 600 nm min^−1^. All measurements were carried out in standard 1 cm path length quartz cuvettes, using triple-distilled water as the analytical blank. Following the Beer–Lambert law, which establishes a linear relationship between absorbance and concentration, the characteristic absorption band at 280 nm was used to monitor chromophore removal. The absorbance at this wavelength served as the quantitative analytical response for estimating the remaining concentration of AELT, as supported by a linear calibration curve (R^2^ > 0.99).

The COD was determined using the closed reflux colorimetric method (Hach Method 8000), which is equivalent to the dichromate digestion method. Briefly, 2.0 mL of the centrifuged supernatant was added to commercial low-range digestion vials (0–150 mg L^−1^). These vials contained a pre-measured solution of potassium dichromate (an oxidizing agent), sulfuric acid, silver sulfate (a catalyst), and mercuric sulfate (to mask chloride interference). The vials were hermetically sealed and digested in a preheated reactor at 150 °C for 120 min. After cooling to room temperature, the absorbance of the samples was measured at 420 nm using a Hach spectrophotometer. COD characterization was performed on the initial AELT dispersion, at the beginning of the dark equilibration period (−30 min), and after 60 min of natural solar irradiation to assess the overall oxidation of the dissolved organic matter.

### 4.5. Kinetic and Statistical Analysis

Prior to solar exposure, the dark adsorption capacity of the catalysts was evaluated. The adsorption efficiency was calculated as a percentage of removal according to Equation (2):

*Adsorption* (%) = [(*C*_−30_ − *C*_0_)/*C*_−30_] × 100 (2)

where *C*_−30_ is the initial concentration (75 mg L^−1^) of the AELT at the beginning of the dark phase (−30 min), and *C*_0_ is the concentration (mg L^−1^) after 30 min of stirring in the absence of light, which corresponds to the start of the solar irradiation (0 min).

The solar-driven chromophore removal efficiency of AELT was calculated as a percentage of removal according to Equation (3):

*Chromophore removal* (%) = [(*C*_0_ − *C_t_*)/*C*_0_] × 100 (3)

where *C*_0_ is the initial concentration (mg L^−1^) of the aqueous extract at the beginning of the irradiation (0 min), and *C_t_* is the concentration (mg L^−1^) at a specific irradiation time *t*. The concentrations were determined from the absorbance values at 280 nm using the previously described calibration curve.

The chromophore removal kinetics of the AELT were evaluated using a pseudo-first-order kinetic model. Extract concentrations were estimated from UV–Vis measurements at 280 nm using a previously established calibration curve prepared from AELT dispersions. The apparent rate constant (*k*) was determined using the pseudo-first-order kinetic model, as shown in Equation (4):

(4) $$C_{t}=C_{0}e^{-kt}$$

where *k* is the chromophore removal constant rate (min^−1^). The equation can be rewritten in its linearized form (Equation (5)) to estimate the rate constant:

(5) $$\ln\left(\frac{C_{t}}{C_{0}}\right)=\;-kt$$

Regression analysis was performed to estimate the *k* value (min^−1^). Finally, the half-life in min (*t*_1/2_) was calculated using Equation (6):

(6) $$t_{1/2}=\frac{ln(2)}{k}$$

The chromophore removal kinetics of AELT were evaluated using the pseudo-first-order kinetic model, applied specifically to the solar irradiation phase (0 to 60 min). To ensure that the kinetic parameters strictly reflect the photochemical transformation, the initial concentration (*C*_0_) was defined as the concentration measured immediately after the 30-min dark equilibration period. The *k* values (min^−1^) was determined by linear regression of the plot versus irradiation time. To maintain mathematical consistency with the model, the regression was performed with the intercept constrained to pass through the origin. The goodness of fit was assessed using the coefficient of determination, and the half-life was calculated to enable a meaningful comparison of chromophore removal rates across the different systems.

All chromophore removal experiments were performed in triplicate. A one-factor experimental design was employed to evaluate the effect of the chromophore removal treatment on process performance. The evaluated factor corresponded to the solar-driven chromophore removal system, with the following levels: solar photolysis, solar/H_2_O_2_, solar/TiO_2_, solar/ZnO, solar/TiO_2_/H_2_O_2_, and solar/ZnO/H_2_O_2_.

Prior to statistical comparisons, data normality was evaluated using the Kolmogorov–Smirnov test. Statistical analyses were subsequently performed using one-way analysis of variance (ANOVA) to determine significant differences among treatments. When statistically significant differences were detected (*p* < 0.05), mean comparisons were carried out using the least significant difference (LSD) test.

## 5. Conclusions

Hydroalcoholic extraction of *L. tridentata* by Soxhlet yielded 26.36%, indicating substantial recovery of solvent-soluble constituents under the applied extraction conditions. Complementary characterization by TGA/DTG, ^1^H/^13^C NMR, FTIR, and GC–MS showed that the hydroalcoholic extract contains a chemically complex mixture of organic constituents with hydroxylated, aromatic, carbonyl, and aliphatic functionalities.

The aqueous hydroalcoholic extract (AELT) exhibited a characteristic absorption maximum at 280 nm, which was used to monitor the transformation of UV-absorbing chromophores during natural-solar treatment. Among the evaluated oxidation systems, Solar/ZnO exhibited the strongest overall performance, achieving the highest reduction in the 280-nm absorbance (89.43 ± 0.69%), the greatest COD removal (88.56 ± 0.01%), the highest apparent pseudo-first-order rate constant (3.67 ± 1.31 × 10^−2^ min^−1^), and the shortest half-life (18.92 ± 0.14 min). In contrast, solar photolysis and Solar/H_2_O_2_ produced comparatively limited transformation of the UV-absorbing constituents.

Overall, the combined UV–Vis, kinetic, and COD results indicate that semiconductor-assisted solar irradiation enhanced the transformation of UV-absorbing constituents and reduced the oxidizable organic load of AELT under the conditions investigated. The results further indicate that ZnO can promote effective solar-driven transformation of complex plant-derived extracts without external H_2_O_2_, whereas the benefit of peroxide addition depended on the semiconductor employed. These findings support the potential of ZnO-assisted natural-solar photocatalysis as a treatment approach for aqueous matrices containing phenolic and other complex phytochemical constituents, while the observed COD reduction should not be interpreted as evidence of complete mineralization.

## Figures and Tables

**Table 1:** Proximate analysis of LT and ELT obtained from thermogravimetric analysis.

Biomass	Moisture (%)	Volatile Solid (%)	Ash (%)
LT *	3.08	91.51	3.82
ELT *	1.90	88.26	2.21

* LT: raw *L. tridentata* biomass; ELT: hydroalcoholic extract of *L. tridentata*.

**Table 2:** ^1^H and ^13^C NMR spectral assignments of the hydroalcoholic extract of *L. tridentata* (ELT) recorded in DMSO-d_6_ and compared with reported literature data for NDGA (δ, ppm).

δ ^1^H DMSO-d_6_	δ ^13^C DMSO-d_6_	* Reported (^1^H) DMSO-d_6_/Acetone-d_6_ **	* Reported (^13^C) DMSO-d_6_/Acetone-d_6_ **
6.62 (d)	115.83 CH	6.62	115.31
6.54 (d)	116.75 CH	6.53	116.23
6.39 (dd)	120.01 CH	6.39	119.52
-	132.75 C	-	132.28
-	145.35 C	-	144.81
-	143.50 C	-	142.96
0.75 (d)	16.57 CH3	0.75	16.05
2.57 (dd)	-	2.70 **	-
2.10 (dd)	38.37 CH2	2.21 **	37.64 **

* Reported data corresponds to NDGA obtained from Spectral Database for Organic Compounds SDBS, National Institute of Advanced Industrial Science and Technology (AIST), Japan, CAS Registry No. 500-38-9. ** Reported in acetone-d_6_. d (doublet), dd (doublet of doublets).

**Table 3:** GC-MS spectral analysis of hydroalcoholic extract of *L. tridentata.*

No.	RT (min)	Compound	Molecular Formula	Molecular Weight	Peak Area %
1	14.98	2-Naphthalenemethanol, decahydro-.α,α.,4a-trimethyl-8-methylene-, [2R-(2.α.,4a.α.,8a.β.)]-	C_15_H_26_O	222.37	1.78
2	17.89	Isophytol	C_20_H_40_O	296.53	0.82
3	18.35	Hexadecanoic acid, ethyl ester	C_18_H_36_O_2_	284.47	23.41
4	19.48	Phytol	C_20_H_40_O	296.53	15.29
5	19.93	9,12-Octadecadienoic acid (Z,Z)-, methyl ester	C_19_H_34_O_2_	294.47	10.32
6	19.99	9,12,15-Octadecatrienoic acid, ethyl ester, (Z,Z,Z)-	C_20_H_34_O_2_	306.48	28.72
7	20.22	Octadecanoic acid, ethyl ester	C_20_H_40_O_2_	312.53	2.12
8	21.1	2-Propenoic acid, 3-(4-hydroxy-3-methoxyphenyl)-	C_10_H_10_O_4_	210.18	8.72
9	28.93	γ-Sitosterol	C_29_H_50_O	414.71	8.75

**Table 4:** Final adsorption and chromophore removal efficiency of AELT after 30 min in the dark and 60 min of solar irradiation under the evaluated solar-driven oxidation systems.

Treatment	Adsorption in Dark (%)	Chromophore Removal Efficiency (%)
Solar	5.12 ± 0.25	8.82 ± 1.67
Solar/H_2_O_2_	4.39 ± 1.55	9.64 ± 2.04
Solar/TiO_2_	9.42 ± 2.61	71.36 ± 1.93
Solar/ZnO	5.51 ± 0.72	89.43 ± 0.69
Solar/TiO_2_/H_2_O_2_	13.76 ± 2.34	84.34 ± 6.86
Solar/ZnO/H_2_O_2_	4.12 ± 1.94	61.32 ± 2.62

**Table 5:** One-way ANOVA results in the chromophore removal efficiencies of AELT under the evaluated solar-driven oxidation systems.

	DF ^1^	Sum of Squares	Mean Square	F-Value	*p*-Value
Treatment	5	19,618.32	3923.66	361.09	1.20 × 10^−12^
Error	12	130.39	10.86		
Total	17	19,748.71			

^1^ Degree of freedom.

**Table 6:** LSD multiple comparison test for the chromophore removal efficiencies of AELT under the evaluated solar-driven oxidation systems.

Treatment	Mean	Groups ^1^		
Solar/ZnO	89.43	A		
Solar/TiO_2_/H_2_O_2_	84.34	A		
Solar/TiO_2_	71.36		B	
Solar/ZnO/H_2_O_2_	61.32		B	
Solar/H_2_O_2_	9.64			C
Solar	8.82			C

^1^ Means that do not share a letter are significantly different.

## Data Availability

The original contributions presented in this study are included in the article. Further inquiries can be directed to the corresponding author.

## Supplementary Materials

The following supporting information can be downloaded at: https://www.mdpi.com/article/10.3390/molecules31162861/s1, Figure S1: Calibration curve relating absorbance at 280 nm to the concentration of the aqueous hydroalcoholic extract of *Larrea tridentata* (AELT); Table S1: Kolmogorov–Smirnov normality test for the chromophore removal efficiencies data.

## Abbreviations

AELT	Aqueous Extract for *Larrea tridentata*
C	Carbon
cm	Centimeters
COD	Chemical organic demand
DMSO	Dimethyl sulfoxide
DTG	Derivative thermogravimetry
ELT	Extract for *Larrea tridentata*
FTIR	Fourier Transform Infrared spectroscopy
g	Grams
GC-MS	Gas Chromatography–Mass Spectrometry
h	Hours
H	Hydrogen
H_2_O_2_	Hydrogen Peroxide
K	Kelvin
L	Liters
LT	*Larrea tridentata*
mg	Milligrams
MHz	MegaHertz
min	Minutes
NDGA	Nordihydroguaiaretic acid
NIST	National Institute Standard and Technology
nm	Nanometers
NMR	Nuclear magnetic resonance
ppm	parts per million
RCF	relative centrifugal force
s	Seconds
SDBS	Spectral Database for Organic Compounds
TGA	Thermogravimetric analysis
TiO_2_	Titanium Dioxide
Uv–vis	UV–visible spectroscopy
v	Volume
ZnO	Zinc Oxide
μL	Microliters
