# Supplementary material for: Physicochemical Characterization and Solar-Driven Chromophore Removal of Hydroalcoholic Larrea tridentata Extracts in Aqueous Media
Source: Molecules. 2026 Aug 16;31(16):2861. doi: 10.3390/molecules31162861 (PMC13515291; doi:10.3390/molecules31162861)
Supplement: Supplementary file 1 [file molecules-31-02861-s001.zip › molecules-4437420-supplementary.pdf]

Article

Physicochemical Characterization and Solar-Driven chromophore removal of Hydroalcoholic *Larrea tridentata* Extracts in Aqueous Media

David Quiroz-Cardoza[1], Juan C. Pantoja Espinoza [1,*], José Béjar [1], José C. Espinoza-Hicks[2], María del Rosario Peralta-Pérez[2], Antonino Pérez-Hernández[1], Francisco Paraguay-Delgado[1] and Víctor M. Orozco-Carmona[1]

[1] Centro de Investigación en Materiales Avanzados SC (CIMAV), Av. Miguel de Cervantes 120, Chihuahua 31136, Chihuahua, México; david.quiroz@cimav.edu.mx, eduardo.garcia@cimav.edu.mx, antonino.perez@gmail.com, francisco.paraguay@cimav.edu.mx, victor.orozco@cimav.edu.mx

[2] Faculty of Chemical Sciences, Autonomous University of Chihuahua, University Circuit S/N, Campus UACH II, Chihuahua 31125, Chihuahua, Mexico; mperalta@uach.mx, jhicks@uach.mx

* Correspondence: juan.pantoja@cimav.edu.mx

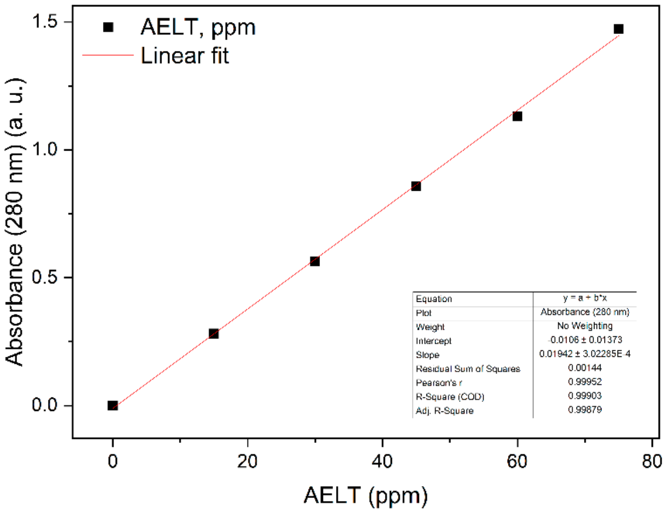

Figure S1. Calibration curve relating absorbance at 280 nm to the concentration of the aqueous hydroalcoholic extract of *Larrea tridentata* (AELT).

Table S1. Kolmogorov–Smirnov normality test for the chromophore removal efficiencies data.

	DF [1]	Statistic	p-value	Decision at level (5%)
Degradation (%)	12	0.22314	0.52578	Can´t reject normality

[1] Degree of freedom.
